# Faecal Calprotectin: A Non-Invasive Marker for Diagnosing and Monitoring Acute Diverticulitis

**DOI:** 10.3390/medsci14030406

**Published:** 2026-07-19

**Authors:** Aamer Mohammed, Yahiya Baig, Alexandra E. Butler

**Affiliations:** 1 School of Medicine, Royal College of Surgeons in Ireland-Medical University of Bahrain, Busaiteen 15503, Bahrain; 21200392@rcsi-mub.com (A.M.); 20206506@rcsi-mub.com (Y.B.); 2 Department of Research, Royal College of Surgeons in Ireland-Medical University of Bahrain, Busaiteen 15503, Bahrain

**Keywords:** acute diverticulitis, diverticular disease, biomarker, faecal calprotectin

## Abstract

Background: Acute diverticulitis (AD) is a prevalent gastrointestinal disorder with a significant recurrence rate. Faecal calprotectin (FC) is a non-invasive biomarker of intestinal inflammation, but its role in diagnosing and monitoring diverticulitis remains to be fully established. Objective: This narrative review aims to evaluate the current evidence on the utility of FC in the diagnosis, severity assessment, prediction of recurrence, and monitoring of therapeutic response in patients with diverticular disease (DD) and AD. Methods: A structured literature search was conducted using PubMed, Scopus, and ScienceDirect for peer-reviewed original studies published in English between 2004 and April 2025. The search strategy combined terms related to diverticular disease and faecal calprotectin. Studies reporting original data on FC in DD were synthesised narratively. Results: FC demonstrates significant utility across multiple clinical applications in DD. For diagnosis, FC is markedly elevated in AD (mean 556–695 μg/g) and symptomatic uncomplicated diverticular disease (SUDD) (median 181 μg/g), while remaining normal in irritable bowel syndrome (mean 50 μg/g), enabling differentiation between organic and functional disorders. FC correlates strongly with endoscopic disease severity, with positivity rates increasing from 48.6% in DICA 1 to 93.2% in DICA 3 (*p* < 0.0001). For predicting recurrence, elevated FC identifies patients at high risk, with one study reporting 87.5% of recurrent cases showing prior FC elevation and a negative predictive value of 96.8%. FC also exhibits excellent short-term prognostic capacity (AUC 0.976 at 3 months) and responds to therapeutic intervention, with significant reductions following successful treatment with probiotics, nutraceuticals, budesonide, and other agents. Conclusions: FC is a promising non-invasive biomarker for diagnosing diverticulitis, assessing disease severity, predicting recurrence, and monitoring treatment response. Its ability to detect subclinical inflammation makes it particularly useful for risk stratification. However, the current evidence base consists predominantly of retrospective and observational studies, and standardised thresholds require further validation through prospective trials before routine clinical implementation can be recommended.

## 1. Introduction

Diverticulitis is a prevalent gastrointestinal disorder defined as macroscopic inflammation of a colonic diverticulum, which can manifest as either uncomplicated disease or complicated cases involving abscess, perforation, stricture, or fistula formation. As a clinical entity, it falls under the broader umbrella of diverticular disease (DD), which encompasses clinically significant and symptomatic diverticulosis resulting from conditions such as diverticulitis, symptomatic uncomplicated diverticular disease (SUDD), and segmental colitis associated with diverticula (SCAD) [1]. The pathophysiology of diverticulitis, while incompletely understood, is increasingly recognized as a chronic inflammatory process, with evidence suggesting that alterations in the gut microbiome including decreased short-chain fatty acid-producing bacteria and compromised mucosal barrier function play a central role alongside established risk factors such as obesity, Western dietary patterns, and genetic predisposition [2]. This modern understanding complements, rather than replaces, the two key traditional theories that primarily explain the initial mechanism of diverticular inflammation: the “traumatic theory,” where faecoliths become entrapped in diverticula, causing mucosal abrasion and bacterial overgrowth, and the “ischaemic theory,” where forceful and prolonged colonic contractions compress the vasculature at the diverticular neck, leading to ischaemia and subsequent micro-perforation [1].

DD exhibits a marked geographic heterogeneity, with the highest incidence observed in Western, developed nations where it affects over 50% of the population above 60 years of age. Its epidemiology is characterized by a rising incidence in younger populations and a dynamic global distribution, increasingly affecting developing regions as they adopt a Westernized lifestyle and diet [3].

Calprotectin is a 36-kDa cytosolic protein derived predominantly from neutrophils that constitutes approximately 60% of their cytosolic protein content, and its concentration in feces is roughly six times higher than in plasma, reflecting its utility as a sensitive marker of intestinal inflammation [4]. Calprotectin comprises two calcium-binding proteins, S100A8 (MRP8) and S100A9 (MRP14), which form a heterodimer that exerts potent antimicrobial properties through the chelation of divalent metal ions essential for bacterial survival [5]. Beyond its antimicrobial function, calprotectin acts as a damage-associated molecular pattern (DAMP) that activates toll-like receptor 4 (TLR4), triggering downstream NF-κB signalling and promoting the release of pro-inflammatory cytokines including interleukin-1β (IL-1β), IL-6, and tumour necrosis factor-α (TNFα) [5,6]. During active inflammation, neutrophil recruitment and NETosis (neutrophil extracellular trap formation) result in substantial calprotectin release into the intestinal lumen, where it can be detected in stool samples [6]. This mechanistic basis explains why FC correlates closely with mucosal neutrophilic infiltrate and why it serves as a sensitive, though non-specific, indicator of intestinal inflammation.

Faecal calprotectin (FC) has emerged as a significantly validated, non-invasive biomarker that allows for the evaluation of gut inflammation, effectively discriminating between inflammatory and non-inflammatory diseases of the intestine. Its concentration in stool correlates significantly with clinical and endoscopic disease activity, particularly in conditions like inflammatory bowel disease, making it an invaluable tool for both initial diagnosis and longitudinal disease monitoring [6]. FC expression has been linked to the severity of DD [7]; however, its role in monitoring the disease after an attack of acute uncomplicated diverticulitis (AUD) is unknown, prompting this review to evaluate whether FC is clinically relevant for predicting relapse.

## 2. Materials and Methods

### 2.1. Search Strategy and Study Selection

A structured literature search was conducted using PubMed, Scopus, and ScienceDirect to identify relevant peer-reviewed original studies published in English between 2004 and April 2025 investigating the role of faecal calprotectin (FC) in the diagnosis and monitoring of diverticulitis. The search strategy combined keywords related to diverticular disease with terms for faecal calprotectin, specifically: (“diverticular disease” OR “diverticulitis” OR “acute diverticulitis” OR “symptomatic uncomplicated diverticular disease” OR “SUDD”) AND (“faecal calprotectin” OR “fecal calprotectin” OR “FC” OR “calprotectin”). Additional references were screened from the bibliographies of selected articles to identify further relevant studies.

### 2.2. Inclusion and Exclusion Criteria

Studies were included if they: (1) investigated the role of FC in patients with diverticular disease or diverticulitis; (2) reported original data on FC levels in relation to diagnosis, disease severity, monitoring of therapeutic response, or prediction of recurrence; (3) were published in peer-reviewed journals in English; and (4) included human subjects. Review articles, editorials, conference abstracts, case reports, and non-peer-reviewed publications were excluded from the primary synthesis but were consulted for background and to identify additional original studies. Studies not published in English or lacking sufficient original data for analysis were also excluded.

### 2.3. Data Extraction

The search identified 65 records from PubMed (*n* = 40), Scopus (*n* = 18), and ScienceDirect (*n* = 7). After removing duplicates (*n* = 12), records were screened by title and abstract (*n* = 53). Non-English publications were excluded (Russian *n* = 3, Italian *n* = 1). Full-text articles were assessed for eligibility (*n* = 49). Of these, 9 reports were excluded: review articles (*n* = 4), a critical appraisal (*n* = 1), case reports (*n* = 3), and a study protocol (*n* = 1). A total of 14 studies met the inclusion criteria and were included in the final analysis.

Data were extracted independently by two authors and included: study design (e.g., prospective cohort, retrospective observational, randomised controlled trial), population characteristics (sample size, age, sex), clinical setting (primary care, emergency department, gastroenterology clinic), FC measurement methods (assay type, thresholds, timing of measurement), comparator groups, and clinical outcomes (diagnostic accuracy, correlation with disease severity, prediction of recurrence, monitoring of treatment response). For studies reporting quantitative data, extracted values included FC concentrations, sensitivity and specificity estimates, hazard ratios, *p*-values, and confidence intervals where available.

## 3. Results

### 3.1. Diagnostic Utility: Differentiating Organic from Functional Disease

**Figure 1:**
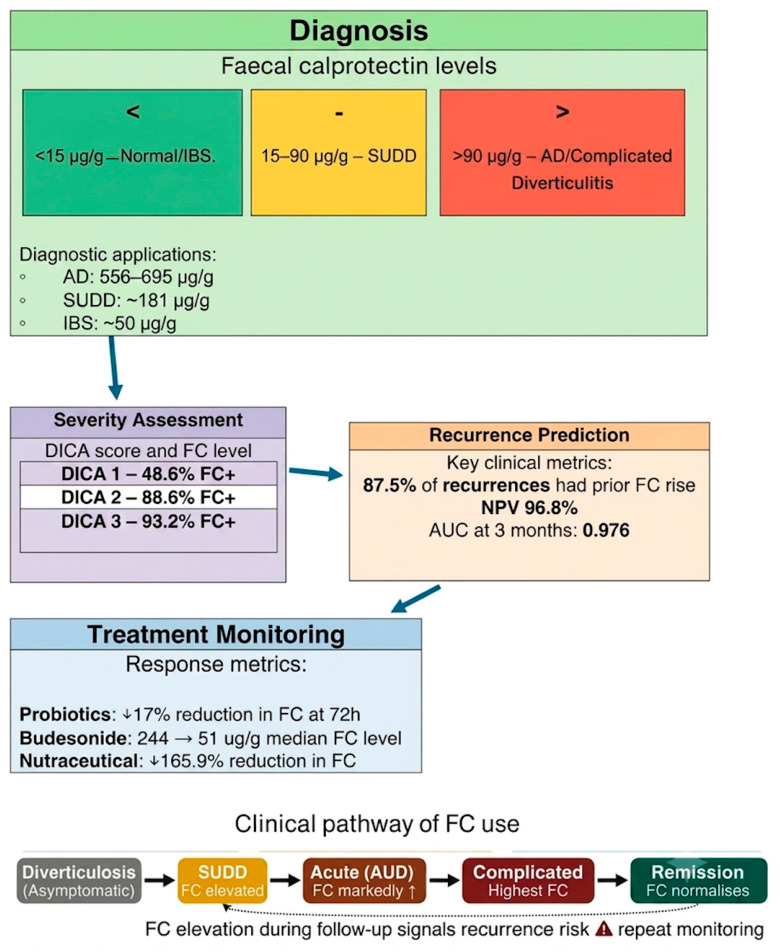
Clinical utility of faecal calprotectin in diverticular disease. The upper panel illustrates the four key clinical roles of faecal calprotectin (FC) in diverticular disease: (1) diagnosis, demonstrating FC elevation across disease subtypes; (2) severity assessment, showing progressive FC positivity with increasing endoscopic severity; (3) recurrence prediction, highlighting the negative predictive value of normal FC during follow-up; and (4) treatment monitoring, demonstrating FC reduction following therapeutic intervention. The green panel displays colour-coded diagnostic FC thresholds. The lower panel depicts the clinical disease continuum from asymptomatic diverticulosis through to remission, with a feedback arrow indicating the role of FC monitoring in identifying recurrence risk. FC concentrations in the diagnostic panel are reported as means or medians depending on the source study. FC positivity rates against endoscopic severity are derived from Tursi, Brandimarte [11]. Recurrence prediction data are derived from Tursi, Elisei [14] and Tursi, Piovani [15]. Treatment monitoring data are derived from Tursi, Cassieri [13], Ojetti, Saviano [17], and Brandimarte, Frajese [18]. Abbreviations: AD, acute diverticulitis; ACD, acute complicated diverticulitis; AUD, acute uncomplicated diverticulitis; AUC, area under the curve; CRP, C-reactive protein; DD, diverticular disease; DICA, Diverticular Inflammation and Complication Assessment; FC, faecal calprotectin; IBS, irritable bowel syndrome; NPV, negative predictive value; SUDD, symptomatic uncomplicated diverticular disease; μg/g, micrograms per gram.

One of the most well-established roles for FC in diverticular disease is its ability to distinguish organic pathology from functional gastrointestinal disorders, particularly irritable bowel syndrome (IBS). This distinction is clinically critical given the overlapping symptom profiles of SUDD and IBS, which have historically posed a diagnostic challenge for clinicians. An overview of the four key clinical applications of FC in diverticular disease is illustrated in Figure 1.

Saviano, Petruzziello [8] studied 146 emergency department patients with abdominal pain and/or diarrhoea, finding 50 (34.24%) had diverticulitis. Mean FC was markedly elevated in acute complicated diverticulitis (ACD) (694.74 ± 315.88 μg/g) and AUD (556.33 ± 330.87 μg/g), while patients with IBS had normal FC (mean 50 ± 47.2 μg/g). For distinguishing AUD from ACD, FC showed a positive predictive value of 40% and negative predictive value of 84%, supporting its use as an emergency triage tool to identify organic pathology warranting further radiological evaluation [8].

Similarly, Kok, Elias [9] evaluated FC in 386 primary care patients with lower abdominal complaints, finding that 75% of diverticulitis patients tested positive on point-of-care FC and 100% on ELISA. The calprotectin ELISA showed sensitivity of 0.74 (95% CI 0.65–0.82). Notably, diverticulosis patients without active inflammation had a median FC of 52 μg/g (POC), suggesting subclinical inflammation in apparently uncomplicated disease [9].

Nevertheless, it must be noted that FC has limitations in the diagnostic context. According to Elias, Kok [10], adding a point-of-care FC test to routine clinical data only marginally increased discriminatory power (AUC rise from 0.741 to 0.763; *p* = 0.078), even though FC alone showed a moderate capacity to distinguish severe colorectal illness (AUC 0.68). Furthermore, combining FC with faecal immunochemical testing (FIT) did not significantly improve model performance beyond FIT alone. This suggests that FC should be interpreted as part of a comprehensive diagnostic algorithm rather than as a standalone test, and that its incremental value may be limited in certain clinical contexts.

### 3.2. Correlation with Endoscopic Disease Severity

Beyond its diagnostic role, FC levels have been shown to correlate meaningfully with the endoscopic severity of diverticular disease, as classified by the Diverticular Inflammation and Complication Assessment (DICA) scoring system. This relationship supports FC as a non-invasive surrogate for endoscopic findings.

Tursi, Brandimarte [11] validated FC against the DICA endoscopic classification in 1651 DD patients. FC positivity was progressively and significantly associated with DICA score: 48.6% in DICA 1 (simple diverticulosis), 88.6% in DICA 2, and 93.2% in DICA 3 (active/past inflammation with complications) (*p* < 0.0001). FC, alongside CRP and abdominal pain, was significantly related to DICA score, supporting FC as a non-invasive surrogate marker for endoscopic severity and for identifying subclinical inflammation in newly diagnosed diverticulosis [11].

Tursi, Piovani [12] confirmed in a cohort of 871 patients that basal FC (median 25 μg/g; range 8–1800 μg/g) was significantly associated with increasing DICA and CODA scores (*p* < 0.001) and with greater bowel habit disturbances (*p* < 0.01). Tursi, Cassieri [13] evaluated patients with advanced DICA 2 and 3 scores who remained symptomatic despite conventional treatment. Following the introduction of budesonide MMX™ (Cosmo Pharmaceuticals SpA, Milan, Italy), median FC levels plummeted from 244.5 µg/g to 51.0 µg/g over six months (*p* < 0.001), effectively reaching the clinical normalisation threshold. This dramatic biochemical response correlated with total symptomatic remission, highlighting FC’s utility in validating the success of alternative anti-inflammatory strategies [13].

### 3.3. Predicting Recurrence and Risk Stratification

Predicting illness recurrence is one of FC’s most useful clinical applications. A study by Tursi, Elisei [14] demonstrated an increased FC in 35.4% of 48 patients monitored for an average of 20 months following an episode of AUD, and 87.5% of patients who experienced recurrence had elevated FC either before or at the time of recurrence. The negative predictive value of 96.8% indicates that a normal FC value during follow-up effectively excludes subsequent relapse, providing clinicians with confidence in identifying low-risk patients who may not require intensive surveillance.

Tursi, Piovani [15] conducted a large prospective cohort study of 871 DD patients followed for three years, finding that each log_10_ increase in FC corresponded to a hazard ratio of 3.29 (95% CI 2.13–5.10; *p* < 0.001) for developing AD. Patients with FC ≥ 90 μg/g had a cumulative AD incidence of 18.9% at three years versus 5.2% for FC < 90 μg/g. While FC showed moderate long-term predictive discrimination (c-statistic 0.685), it demonstrated excellent short-term prognostic capacity, with time-dependent AUC of 0.976 at three months, comparable to the validated CODA score (AUC 0.962). Adding FC to the DICA classification improved the c-statistic from 0.779 to 0.806 (*p* = 0.022), though adding FC to CODA did not significantly improve long-term prediction [15].

An observational prospective study conducted by Lahat, Necula [16] investigated 16 patients following an episode of AD, stratifying patients into those with previous severe AD (n = 8) defined by radiographic criteria (abscess, extraluminal air, or extraluminal contrast on CT) and those with nonsevere AD (n = 8). At a median of 12 weeks following the acute episode, patients with previous severe AD had mean FC levels more than three times higher than those with nonsevere AD (115.75 ± 85.9 μg/g vs. 35 ± 8.74 μg/g), though this difference did not reach statistical significance (*p* = 0.08), likely due to the small sample size. Clinically, all patients with previous severe AD reported persistent symptoms fulfilling criteria for SUDD, whereas none of the nonsevere AD patients experienced such symptoms (*p* = 0.0002). These elevated FC levels in the severe AD group correlated with significantly higher tissue expression of pro-inflammatory cytokines TNFα and IL-6 in diverticular mucosa (*p* = 0.0045 and *p* = 0.0084, respectively) and with histologic evidence of chronic inflammation including crypt distortion, lymphoid aggregates, and increased lymphocytic infiltration. The study demonstrates that FC, as a non-invasive marker, reflects the ongoing low-grade mucosal inflammation that persists after severe AD episodes and underlies chronic symptoms, supporting its utility in identifying patients who may benefit from closer surveillance or anti-inflammatory therapy [16].

### 3.4. Monitoring Therapeutic Response

FC has also proven valuable as an objective measure of treatment efficacy across a range of therapeutic interventions for diverticular disease, from probiotics and nutraceuticals to corticosteroids.

Ojetti, Saviano [17] randomised 119 AUD patients to Limosilactobacillus reuteri ATCC PTA 4659 or placebo alongside standard treatment. At enrolment, both groups had markedly elevated FC (probiotic group: 640.01 ± 150.20 mg/L; placebo: 568.20 ± 130.65 mg/L). After 72 h, the probiotic group showed a 17% reduction versus 10.6% in the placebo group (*p* < 0.05), confirming FC as a sensitive marker of therapeutic response in AUD and demonstrating that probiotics accelerate mucosal inflammation resolution. The modest absolute reduction at 72 h reflects the slower resolution of tissue-level inflammation compared to systemic markers such as CRP [17].

Brandimarte, Frajese [18] evaluated FC as a marker in 350 SUDD patients receiving a nutraceutical formulation (Enteroflegin^®^, Manufacturer: Fenix Pharma Soc. Coop. p.A., Rome, Italy). Median FC decreased from 181.3 μg/g at baseline to 100.2 μg/g at 3 months (44.8% reduction; *p* < 0.001) and 67.9 μg/g at 6 months (65.9% reduction; *p* < 0.001), paralleling significant reduction in abdominal pain VAS scores. Higher baseline FC and greater symptom burden predicted greater FC reduction. Only 2% of patients developed AD during the study, all with DICA 2–3 scores at baseline [18].

Tursi, Mocci [19] evaluated FC in 24 moderate-to-severe DD patients treated with a symbiotic mixture (Prolactis GG Plus^®^, Manufacturer: Omega Pharma S.p.A., Cantù, Italy). FC decreased significantly in DICA 2 patients (from 112 ± 30 to 61 ± 9 μg/g; *p* < 0.02), while remaining unchanged in DICA 3 patients (126 ± 22 μg/g; *p* = 0.132). Two DICA 3 patients with rising FC (194 ± 15 μg/g) subsequently developed AUD. These findings suggest that persistently elevated FC in severe disease identifies patients at risk for complications requiring additional therapeutic intervention [19].

### 3.5. Microbiota Correlations

Evidence suggests that FC levels reflect both mucosal inflammation and gut microbiota dysbiosis, providing a mechanistic link between microbial alterations and the inflammatory burden detectable by FC. Kvasnovsky, Leong [20] found in 28 SUDD patients that FC positively correlated with alpha microbial diversity (Shannon index, *p* = 0.005) and Lactobacillus abundance (*p* = 0.004), suggesting a compensatory microbial response to inflammation. Mean FC was higher in patients with prior AD history (104.1 ± 139.2 μg/g) versus those without (48.5 ± 52.0 μg/g), though not statistically significant (*p* = 0.18) [20].

### 3.6. Important Confounders and Limitations in the Literature

These findings should be interpreted in light of certain limitations in the current literature. Significant heterogeneity in study designs ranging from retrospective cohorts to randomized controlled trials and small sample sizes, such as those noted by Lahat, Necula [16] and Tursi, Piovani [12], often preclude meta-analysis and limit statistical power. Furthermore, a lack of standardized FC measurement protocols, varying diagnostic thresholds (50–100 μg/g), and inconsistent classification of DD subtypes (e.g., the SUDD-IBS overlap) complicate cross-study comparisons.

Critically, many analyses fail to account for pharmacological confounders. In a retrospective study of 585 patients with normal colonoscopy findings who underwent FC testing, Hovstadius, Lundgren [21] found that among patients with FC > 50 μg/g (34% of the cohort), there was a higher prevalence of colonic diverticulosis compared to those with normal FC (41% vs. 35%; *p* = 0.015). During three-year follow-up, AD was diagnosed in 13 patients, though only 4 (31%) had elevated FC at baseline. Patients with elevated FC were more likely to be on proton pump inhibitors (63% vs. 26%; *p* < 0.001), acetylsalicylic acid (60% vs. 28%; *p* < 0.001), and NSAIDs (54% vs. 33%; *p* = 0.003), highlighting important confounders. While elevated FC is associated with diverticulosis, a normal colonoscopy in a patient with raised FC does not predict increased risk of developing diverticulitis during follow-up [21]. A summary of all included studies, including their design, population, key FC findings, and clinical applications, is presented in Table 1.

## 4. Discussion

The current narrative synthesis reveals that FC holds considerable promise as a non-invasive biomarker across the clinical spectrum of diverticular disease, from initial diagnosis through to longitudinal monitoring and recurrence prediction. However, the evidence base is characterised by important methodological limitations that must temper enthusiasm for immediate clinical implementation. 

### 4.1. Diagnostic Utility and Proposed Thresholds

The available evidence supports a hierarchical relationship between FC concentrations and the severity of diverticular inflammation. Tursi [9] proposed that FC values ≥ 60 μg/g are characteristic of AUD, while values ≤ 15 μg/g are typical of IBS patients and healthy controls. This stratification is supported by empirical data: Saviano et al. [8] reported mean FC of 556.33 ± 330.87 μg/g in AUD and 50 ± 47.2 μg/g in IBS, while Brandimarte et al. [18] observed a median FC of 181.3 μg/g in SUDD. Table 2 summarises the evidence supporting these proposed thresholds. However, these thresholds derive predominantly from single-centre studies with modest sample sizes and require independent validation. The considerable overlap in FC values between disease states—evidenced by the wide ranges reported (e.g., 8–1800 μg/g in newly diagnosed DD [12])—limits the diagnostic precision of any single cut-off. Clinicians should therefore interpret FC values as part of a comprehensive assessment incorporating clinical history, symptom profile, and imaging findings, rather than relying on isolated biomarker results.

While FC is valuable for distinguishing diverticular disease from IBS, clinicians must recognise that FC is a non-specific marker of intestinal inflammation. Elevated FC may be observed in several other gastrointestinal conditions that can present with similar symptoms or coexist with diverticular disease. Inflammatory bowel disease (IBD), particularly Crohn’s disease, frequently presents with FC levels exceeding 200–600 μg/g, often higher than those seen in uncomplicated diverticulitis. Microscopic colitis, an increasingly recognised cause of chronic watery diarrhoea in older adults, can produce FC levels comparable to those seen in acute diverticulitis [22]. Colorectal neoplasia, including advanced adenomas and carcinomas, may cause modest FC elevation through mucosal disruption and associated inflammation. Infectious colitis typically produces transient but marked FC elevation that resolves with infection clearance. Segmental colitis associated with diverticula (SCAD), a distinct inflammatory condition affecting the inter-diverticular mucosa, may be indistinguishable from diverticulitis based on FC alone and requires endoscopic confirmation.

### 4.2. Correlation with Disease Severity

The progressive increase in FC positivity across DICA classifications—from 48.6% in DICA 1 to 93.2% in DICA 3 [11]—establishes a clear relationship between FC and endoscopic severity. This correlation is biologically plausible: calprotectin is released during neutrophil activation and NETosis, processes that intensify with increasing mucosal inflammation. The S100A8/S100A9 heterodimer (calprotectin) binds to TLR4, amplifying pro-inflammatory signalling through NF-κB and promoting further neutrophil recruitment via IL-1β and IL-17 pathways [5,6]. In diverticular disease, this inflammatory cascade is driven by microbiota dysbiosis, epithelial barrier dysfunction, and faecolith-induced mucosal injury [1,2]. The ability of FC to detect subclinical inflammation in DICA 1 patients (48.6% positivity) is particularly noteworthy, as it identifies individuals with radiologically uncomplicated diverticulosis who may nonetheless harbour active mucosal disease.

### 4.3. Predicting Recurrence: Promise and Limitations

The predictive capacity of FC for diverticulitis recurrence represents one of its most clinically compelling applications. The negative predictive value of 96.8% reported by Tursi et al. [14] suggests that a normal FC during follow-up effectively excludes subsequent relapse, enabling confident identification of low-risk patients. The short-term prognostic discrimination is excellent (AUC 0.976 at 3 months) [15], comparable to validated clinical scores such as CODA. However, the long-term predictive performance is more modest (c-statistic 0.685), indicating that FC alone cannot reliably stratify risk beyond the immediate post-acute period. This temporal limitation likely reflects the dynamic nature of mucosal inflammation, which may fluctuate with dietary changes, medication use, and microbiota shifts. The optimal timing and frequency of FC measurement for recurrence prediction remain undefined; current protocols vary from single measurements to serial testing at 3, 6, and 12-month intervals [14,15,20].

### 4.4. Therapeutic Monitoring

FC responds dynamically to therapeutic intervention, with significant reductions documented following probiotic, nutraceutical, and corticosteroid therapy [13,17,18]. This responsiveness supports its utility as an objective treatment efficacy marker. However, the clinical significance of FC reduction varies by context: in AUD, a 17% reduction at 72 h [17] may reflect early anti-inflammatory effects, while the 65.9% reduction at 6 months in SUDD [18] likely represents resolution of chronic low-grade inflammation. The observation that DICA 3 patients showed no significant FC reduction with symbiotic therapy and subsequently developed acute diverticulitis [19] underscores that FC non-response may identify treatment failure and impending complications.

### 4.5. Comparison with Competing Biomarkers

Table 3 summarises the relative performance of FC against established and emerging biomarkers in diverticular disease. C-reactive protein (CRP) and white cell count (WCC) are widely available and well-validated for acute diagnosis but lack specificity for intestinal inflammation and show poor correlation with endoscopic severity or recurrence risk. Faecal immunochemical testing (FIT) demonstrates high sensitivity for colorectal neoplasia but has no established role in diverticulitis diagnosis or monitoring. Procalcitonin and lactoferrin remain investigational in this context. Imaging modalities (CT, ultrasound) provide anatomical detail essential for complicated disease but are invasive, expensive, and impractical for longitudinal monitoring. FC occupies a unique position: it is non-invasive, stool-based, correlates with mucosal inflammation, and enables repeated measurement. However, its non-specificity—elevated levels occur in IBD, microscopic colitis, infectious colitis, and with NSAID/PPI use [13,21]—remains a critical limitation.

### 4.6. Clinical Implementation: A Cautious Approach

From a clinical practice perspective, integrating FC into diverticulitis management requires applying its strengths without overinterpreting isolated results. For diagnosis, an FC ≥ 60 μg/g in a patient with lower abdominal pain should prompt investigation for organic pathology (particularly AUD), while an FC ≤ 15 μg/g strongly suggests a functional disorder like IBS. However, FC elevation is non-specific and must be interpreted alongside clinical history, symptom profile, and medication use (NSAIDs, aspirin, PPIs). For treatment monitoring, serial FC measurements provide objective evidence of therapeutic response: a declining trajectory supports continuation of the current regimen, while a persistently elevated or rising FC, particularly above 90 μg/g, should prompt clinical reassessment or earlier endoscopic evaluation. For recurrence risk stratification, clinicians should adopt longitudinal FC trajectories rather than relying on isolated values; a normal FC during follow-up offers high negative predictive value, enabling confident deferral of invasive investigations in low-risk patients.

It must be emphasised that these recommendations are based on observational and retrospective data of variable quality. The paucity of large, prospective, multicentre trials limits the strength of evidence for clinical implementation. Clinicians should exercise caution in applying FC-based algorithms until prospective validation—such as that planned in the DICRO trial [20]—demonstrates reproducible thresholds and cost-effective outcomes.

### 4.7. Future Directions

Several priorities emerge for advancing the clinical utility of FC in diverticular disease. First, standardisation of measurement protocols and harmonisation of diagnostic thresholds across assay platforms are essential. Second, the ongoing DICRO trial [20] will provide much-needed prospective validation of FC as a prognostic biomarker. Third, mechanistic studies elucidating the relationship between FC elevation, specific microbiota alterations, and mucosal immune responses could inform targeted therapeutic strategies. Fourth, cost-effectiveness analyses comparing FC-based monitoring against standard care are required to justify reimbursement and clinical adoption. Finally, the development of combined biomarker panels incorporating FC, CRP, and microbiome signatures may improve diagnostic and prognostic accuracy beyond any single marker.

## 5. Conclusions

This review confirms that FC is a promising non-invasive biomarker with potential applications in diagnosing diverticulitis, assessing disease severity, predicting recurrence, and monitoring treatment response. Its ability to detect subclinical inflammation and its excellent short-term prognostic capacity make it an attractive tool for risk stratification. However, the current evidence base is limited by retrospective study designs, small sample sizes, lack of standardised thresholds, and important pharmacological confounders. FC should not be viewed as a standalone diagnostic test but rather as a complementary tool within a comprehensive clinical assessment. Its integration into routine management awaits validation through prospective trials such as the DICRO trial, which will be pivotal in establishing evidence-based thresholds and refining management strategies for patients with diverticular disease.

## Figures and Tables

**Table 1:** Summary of Studies Investigating Faecal Calprotectin in Diverticular Disease.

Reference	Study Design	Sample Size	Population	Key FC Findings	Clinical Application
Saviano, Petruzziello [8]	Retrospective observational	146 patients	ED patients with abdominal pain	ACD: 694.74 ± 315.88 μg/g; AUD: 556.33 ± 330.87 μg/g; IBS: 50 ± 47.2 μg/g; PPV 40%, NPV 84% for AUD vs. ACD	Diagnosis in emergency setting
Kok, Elias [9]	Prospective diagnostic (CEDAR)	386 patients	Primary care with lower abdominal complaints	Diverticulitis patients: median POC FC 220 μg/g, ELISA 477 μg/g	Diagnosis in primary care
Elias, Kok [10]	Prospective cross-sectional diagnostic study	810 primary care patients	Patients suspected of significant colorectal disease (including 18 with diverticulitis)	Adding quantitative POC calprotectin to clinical data increased AUC from 0.741 to 0.763 (*p* = 0.078)—not statistically significant	Diagnosis in primary care; limited utility as standalone test; better performance when combined with FIT
Tursi, Brandimarte [11]	Retrospective cohort	1651 patients	DD patients with DICA classification	FC positivity: DICA 1: 48.6%, DICA 2: 88.6%, DICA 3: 93.2% (*p* < 0.0001)	Correlating with endoscopic severity
Tursi, Piovani [12]	Multicenter prospective cohort	871 patients	Newly diagnosed DD	FC range 8–1800 μg/g, median 25 μg/g; higher FC with constipation/diarrhoea (*p* < 0.01); FC > 90 μg/g: 18.9% AD incidence	Risk stratification, correlation with symptoms
Tursi, Cassieri [13]	Post-hoc analysis	24 patients	DICA 2/3 patients, treatment-resistant	Baseline FC 244.5 µg/g → 51.0 µg/g at 6 months (*p* < 0.001) post-budesonide; normalised with clinical remission	Monitoring therapeutic response
Tursi, Elisei [14]	Prospective cohort	54 patients (48 analysed)	AUD patients after remission	Increased FC detected in 35.4% during follow-up; 87.5% of recurrence cases had elevated FC; NPV 96.8% for recurrence	Predicting recurrence
Tursi, Piovani [15]	International prospective cohort	871 patients	Newly diagnosed DD, 3-year follow-up	HR 3.29 per log increase; FC ≥ 90 μg/g: 18.9% AD incidence vs. 5.2% with lower FC; 3-month AUC 0.976	Predicting AD, short-term risk stratification
Lahat, Necula [16]	Prospective observational	16 patients	Post-AD (8 severe, 8 nonsevere)	Severe AD: 115.75 ± 85.9 μg/g; nonsevere: 35 ± 8.74 μg/g (*p* = 0.08); correlated with cytokine expression	Persistent inflammation after severe AD
Ojetti, Saviano [17]	RCT	119 patients	AUD patients	Baseline FC: 640.01 ± 150.20 mg/L (probiotic), 568.20 ± 130.65 mg/L (placebo); 72 h reduction: 17% vs. 10.6% (*p* < 0.05)	Monitoring therapeutic response
Brandimarte, Frajese [18]	Retrospective observational	350 patients	SUDD on nutraceutical treatment	Baseline FC 181.3 μg/g → 100.2 μg/g at 3 months (44.8% reduction, *p* < 0.001) → 67.9 μg/g at 6 months (65.9% reduction, *p* < 0.001)	Monitoring therapeutic response
Tursi, Mocci [19]	Retrospective	24 patients	DICA 2/3 on symbiotic treatment	Baseline FC: DICA 2: 112 ± 30 μg/g, DICA 3: 126 ± 22 μg/g (*p* = 0.05). DICA 2 FC decreased significantly (*p* < 0.02); DICA 3 FC unchanged (*p* = 0.132). Two DICA 3 patients with rising FC (194 ± 15 μg/g) developed AUD.	Monitoring treatment response by severity
Kvasnovsky, Leong [20]	Pilot study	28 patients	SUDD patients	FC correlated with alpha diversity (*p* = 0.005) and Lactobacillus abundance (*p* = 0.004); previous AD: 104.1 ± 139.2 μg/g vs. no AD: 48.5 ± 52.0 μg/g	Microbiota correlations
Hovstadius, Lundgren [21]	Retrospective observational	585 patients	Normal colonoscopy, FC tested	FC > 50 μg/g: 34% of cohort; diverticulosis prevalence 41% vs. 35% (*p* = 0.015); 13 AD cases, only 31% had elevated FC at baseline	Association with diverticulosis, confounders

**Table 2:** Evidence Summary for Proposed Faecal Calprotectin Diagnostic Thresholds in Diverticular Disease.

Proposed Threshold	Clinical Interpretation	Supporting Evidence	Key Limitations
≤15 μg/g	Normal/healthy controls; typical of IBS	Tursi [7]; Saviano et al. [8] (IBS mean 50 ± 47.2 μg/g, but IBS subgroup may be lower)	Overlap with quiescent DD; some healthy individuals have FC up to 50 μg/g
~15–60 μg/g	SUDD; subclinical inflammation	Tursi [7]; Brandimarte et al. [18] (median 181.3 μg/g at baseline, decreasing to 67.9 μg/g); Kok et al. [9] (diverticulosis without active inflammation: median 52 μg/g)	Wide range; significant overlap with IBS and healthy controls; no validated cut-off
≥60 μg/g	AUD; active diverticular inflammation	Tursi [7]; Saviano et al. [8] (AUD mean 556.33 ± 330.87 μg/g); Ojetti et al. [17] (baseline ~640 mg/L ≈ ~640 μg/g)	Considerable inter-individual variability; non-specific (also elevated in IBD, infectious colitis, microscopic colitis)
≥90 μg/g	High risk for AD recurrence	Tursi et al. [15] (18.9% vs. 5.2% 3-year AD incidence)	Derived from single prospective cohort; requires external validation
>50 μg/g	Associated with diverticulosis; pharmacological confounders common	Hovstadius et al. [21]	High rates of PPI, aspirin, and NSAID use in this group limit specificity

**Table 3:** Comparison of Biomarkers and Diagnostic Modalities in Diverticular Disease.

Marker/Modality	Diagnosis	Severity Assessment	Recurrence Prediction	Treatment Monitoring	Key Advantages	Key Limitations
FC	Good	Good	Good (short-term)	Good	Non-invasive; stool-based; correlates with mucosal inflammation; repeatable	Non-specific; affected by medications (PPIs, NSAIDs, aspirin); no standardised thresholds
CRP	Good	Moderate	Poor	Moderate	Widely available; rapid turnaround; well-validated for acute inflammation	Lacks intestinal specificity; normalises quickly; poor correlation with endoscopic severity
WCC	Moderate	Poor	Poor	Poor	Routine test; inexpensive	Very non-specific; poor prognostic value
FIT	Limited	None	None	None	Highly sensitive for colorectal neoplasia; inexpensive	No role in diverticulitis; false positives with inflammation
CT imaging	Excellent (complicated)	Excellent	N/A	N/A	Gold standard for complicated disease; anatomical detail	Radiation exposure; expensive; not repeatable for monitoring
Ultrasound	Moderate	Moderate	N/A	N/A	No radiation; inexpensive; bedside availability	Operator-dependent; limited in obese patients; poor for deep structures

## Data Availability

No new data were created or analyzed in this study. Data sharing is not applicable to this article.

## Abbreviations

The following abbreviations are used in this manuscript:

AD	acute diverticulitis
ACD	acute complicated diverticulitis
AUD	acute uncomplicated diverticulitis
AUC	area under the curve
CI	confidence interval
CODA	a validated prognostic score for diverticular disease
CRP	C-reactive protein
CT	computed tomography
DAMP	damage-associated molecular pattern
DD	diverticular disease
DICA	Diverticular Inflammation and Complication Assessment
ELISA	enzyme-linked immunosorbent assay
FC	faecal calprotectin
FIT	faecal immunochemical testing
GIQLI	Gastrointestinal Quality of Life Index
IBD	inflammatory bowel disease
IBS	irritable bowel syndrome
IL-6	interleukin-6
IQR	interquartile range
kDa	kilodalton
NPV	negative predictive value
NSAID	non-steroidal anti-inflammatory drug
POC	point-of-care
PPV	positive predictive value
PRISMA	Preferred Reporting Items for Systematic Reviews and Meta-Analyses
RCT	randomised controlled trial
SCAD	segmental colitis associated with diverticula
SF-12	Short Form 12 Health Survey
SUDD	symptomatic uncomplicated diverticular disease
TNFα	tumour necrosis factor alpha
VAS	visual analogue scale
μg/g	micrograms per gram
